# New Cembranolides from the Dongsha Atoll Soft Coral *Lobophytum durum*

**DOI:** 10.3390/md9081307

**Published:** 2011-07-27

**Authors:** Shi-Yie Cheng, Pei-Wen Chen, Hwa-Pyng Chen, Shang-Kwei Wang, Chang-Yih Duh

**Affiliations:** 1 Department of Marine Biotechnology and Resources, National Sun Yat-sen University, Kaohsiung 804, Taiwan; E-Mails: shiyie63@yahoo.com.tw (S.-Y.C.); m975020018@student.nsysu.edu.tw (P.-W.C.); m975020801@student.nsysu.edu.tw (H.-P.C.); 2 Department of Microbiology, Kaohsiung Medical University, Kaohsiung 807, Taiwan; 3 Center for Asia-Pacific Ocean Research and Translational Biopharmaceuticals, National Sun Yat-sen University, Kaohsiung 804, Taiwan

**Keywords:** soft coral, *Lobophytum durum*, cytotoxicity, antiviral activity

## Abstract

Chemical investigations of the Dongsha Atoll soft coral *Lobophytum durum* resulted in the isolation of five new cembranolides, durumolides M–Q (**1**–**5**). The structures of compounds **1**–**5** were characterized by the interpretation of extensive spectroscopic analysis. Compound **4** exhibited cytotoxicity against P-388 (mouse lymphocytic leukemia) cell line with an ED_50_ of 3.8 μg/mL. Moreover, compound **5** showed significant antiviral activity against human cytomegalovirus with an IC_50_ of 5.2 μg/mL.

## 1. Introduction

**Figure 1.**
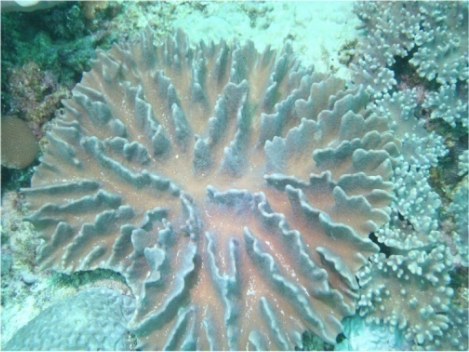
Soft coral *Lobophytum durum*

**Figure 2.**
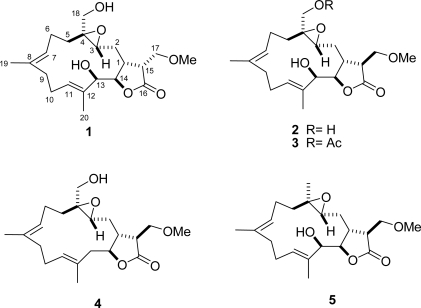
Structures of compounds **1**–**5**.

Various natural products, distributed mainly in marine soft corals of the genus *Lobophytum* (Alcyoniidae) [1–21], have attracted much attention from chemists specializing in natural products due to their structural complexity and remarkable pharmacological activities such as cytotoxicity [2–9], antibacterial activities [10], anti-inflammatory properties [10–12], and HIV-inhibitory activity [13]. Our previous investigations of the soft coral *L. durum* (Figure 1) from the Dongsha Atoll have resulted in the purification of 15 cembranolides, durumolides A–L and durumhemiketalolides A–C, several of which showed antibacterial and anti-inflammatory activities [10–12]. Our continuing chemical examinations of the bioactive substances of this organism led to the isolation of five new cembranolides, designated as durumolides M–Q (**1**–**5**) (Figure 2). The structures of compounds **1**–**5** were determined on the basis of detailed 1D and 2D NMR experiments, mainly employing COSY, DEPT, HMBC, HSQC, and NOESY spectra. Moreover, durumolides M–Q were evaluated *in vitro* for the cytotoxicity against A-459 (human lung adenocarcinoma), HT-29 (human colon adenocarcinoma), and P-388 (mouse lymphocytic leukemia) cancer cell lines, and antiviral activity against human cytomegalovirus.

## 2. Results and Discussion

Specimens of *L. durum* were frozen immediately after collection. Conventional extraction procedures were used, and the acetone extract was exhaustively partitioned between EtOAc and H_2_O to afford the EtOAc-soluble fraction, which was evaporated under vacuum to yield a dark brown gum (30 g). The concentrated residue was subjected to column chromatography and high-performance liquid chromatography (HPLC), leading to the purification of **1**–**5**.

**Figure 3.**
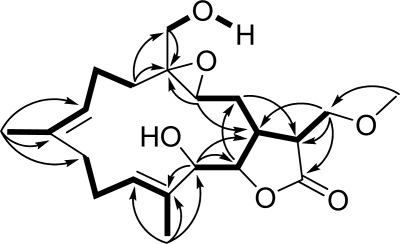
Selected ^1^H–^1^H COSY (▬) and HMBC (→) correlations of **1**.

Durumolide M (**1**), appeared as a colorless oil, exhibited a high resolution electrospray ionization mass spectrometry (HR-ESI-MS) pseudomolecular ion peak at *m*/*z* 403.2099 [M + Na]^+^, corresponding to a molecular formula of C_21_H_32_O_6_ and six degrees of unsaturation. Comparison of the NMR data (Tables 1 and 2) of **1** with those of sinularolide B [22] revealed that **1** was determined to be a 17-methoxylated analogue of sinularolide B, coinciding with methoxyl protons at *δ*_H_ 3.30 (3H, s) correlated to the oxymethine carbon at *δ*_C_ 70.0 (C-17), and oxymethylene protons at *δ*_H_ 3.81 (1H, dd, *J* = 9.2, 2.8 Hz, H-17a) and 3.73 (1H, dd, *J* = 9.2, 2.8 Hz, H-17b) correlated to the methine carbons at *δ*_C_ 40.8 (C-1), and 44.5 (C-15), as well as the lactone carbonyl carbon at *δ*_C_ 176.9 (C-16) in the HMBC spectrum (Figure 3). Thus, the planar structure of durumolide M, possessing an α-methoxymethyl-γ-lactone ring fused to a 14-membered ring at C-1 and C-14, was, with certainty, assigned as **1**.

**Figure 4.**
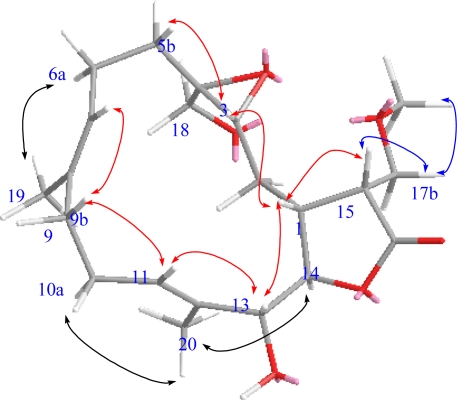
Selected NOESY correlations of **1**.

The geometries of the two trisubstituted olefins were proposed as both *E* based on the γ-effect of the olefinic methyl signals for C-19 and C-20 (less than 20 ppm) and the NOESY correlations between H-7/H-9b and H-11/H-13 (Figure 4). Compound **1** possessed the same configurations as sinularolide B at the C-1, C-3, C-4, C-13, and C-14 stereocenters due to the similar NOESY correlations between H-1/H-3, H-1/H-13, H-11/H-13, H-11/H-9b (*δ* 2.13), H-3/H-5b (*δ* 1.11), H-14/H-2a (*δ* 1.75), H-7/H-9b, and H-14/H_3_-20. Moreover, the large coupling constant (*J* = 9.2 Hz) between H-1 and H-15 suggested that the vicinal protons were either in an anticoplanar or eclipse relationship. The latter relationship should be correct because the signal at *δ*_H_ 2.86 (1H, dt, *J* = 9.2, 2.8 Hz, H-15) showed a strong NOESY correlation with *δ*_H_ 2.28 (1H, m, H-1), implying the *R* configuration at C-15. On the basis of the above-mentioned observations, the structure of durumolide M (**1**) was characterized as (1R*,3R*,4S*, 13R*,14R*,15R*,7*E*,11*E*)-13,18-dihydroxy-17-methoxy-3,4-epoxycembra-7,11-dien-16,14-olide.

**Figure 5.**
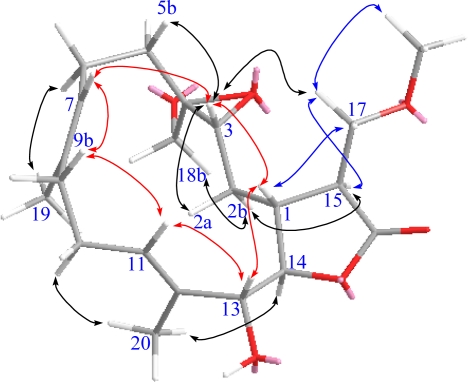
Selected NOESY correlations of **2**.

Compounds **1** and **2** had the same molecular formula (C_21_H_32_O_6_). Analysis of their ^1^H- and ^13^C-NMR data (Tables 1 and 2) revealed that they shared the same cembranolide skeleton, differing only in the configuration of C-15 in **2**. The configuration of C-15 was proposed to be *S*, as identified by its NOESY correlations between H-15/H-2b, H-14/H-2b, and H_2_-17/H-1 (Figure 5). Therefore, compound **2** was determined to be 15-epimer of **1**. According to the above findings, the structure of durumolide N (**2**) was unambiguously established and elucidated as (1R*,3R*,4S*,13R*,14R*, 15S*,7*E*,11*E*)-13,18-dihydroxy-17-methoxy-3,4-epoxycembra-7,11-dien-16,14-olide.

Durumolide O (**3**) was analyzed for a molecular formula of C_23_H_34_O_7_ by its HR-ESI-MS coupled with the DEPT and ^13^C NMR spectroscopic data (Table 2). Analysis of the ^1^H–^1^H COSY and HMBC correlations were diagnostic in determining that the planar framework of durumolide O, having a 14-membered ring fused to an α-methoxymethyl-γ-lactone ring at C-1 and C-14, was proposed as **3**. The structure of **3** was identical to that of **2** except that the hydroxy group attached to C-18 was replaced by an acetoxy group. This assumption was confirmed through the key HMBC correlations from H_2_-18 to C-3, C-4, C-5, and the carbonyl carbon of 18-OAc. All the NMR data (Tables 1 and 2) of **3**, assigned by COSY, HMBC, HSQC, and NOESY correlations, were satisfactorily consistent with the structure shown as (1R*,3R*,4S*,13R*,14R*,15S*,7*E*,11*E*)-13-hydroxy-18-acetoxy-17-methoxy-3,4-epoxycembra-7,11-dien-16,14-olide.

The positive HR-ESI-MS of durumolide P (**4**) exhibited a pseudomolecular ion peak at *m*/*z* 387.2148 [M + Na]^+^, consistent with a molecular formula of C_21_H_32_O_5_, which is 16 mass units smaller than that of **1**. Comparison of the NMR data (Tables 2 and 3) of both compounds showed that **4** exhibited the same framework of an α-methoxymethyl-γ-lactone-containing cembranolide as **2**, with the exception of signals assigned to C-13, where the oxymethine in **2** was replaced by a methylene [*δ*_H_ 2.54 (1H, m) and 2.45 (1H, m); *δ*_C_ 43.7 (CH_2_)] in **4**. The observed COSY correlation between H-13 and H-14 and the key HMBC correlations from H_3_-20 to C-13 confirmed the location of the methylene group. The relative stereochemistry of **4** was in agreement with that of **2** due to the similar NOESY correlations. Judging from the undisputable evidence, the structure of **4** was unambiguously determined and deduced as (1R*,3R*,4S*,14S*,15S*,7*E*,11*E*)-18-hydroxy-17-methoxy-3,4-epoxycembra-7,11-dien-16,14-olide.

**Figure 6.**
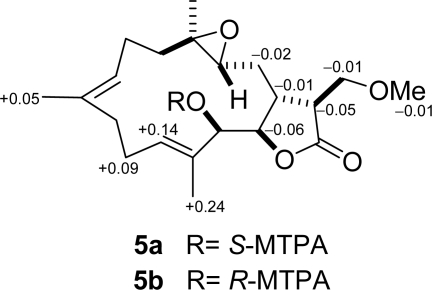
^1^H-NMR chemical shift differences (Δ*δ* = *δ_S_* − *δ_R_*) of the MTPA esters in pyridine-*d*_5_.

The molecular formula of C_21_H_32_O_5_ was assigned to **5** from its HR-ESI-MS and ^13^C NMR data (Table 2), indicating six degrees of unsaturation. The NMR data (Tables 2 and 3) of **5** were highly compatible with those obtained for durumolide J [11], with the replacement of the α-methylene-γ-lactone with an α-methoxymethyl-γ-lactone being the most noticeable difference. The methoxymethyl moiety attached to C-15 was inferred from the ^1^H–^1^H COSY and HMBC correlations. Moreover, the 15*S* configuration was confirmed by the key NOESY correlation between H-17/H-1 and H-1/H-3. The appropriate stereochemistry of **5** was determined by spectroscopic method according to Mosher’s acylation for absolute configuration determination of chiral alcohols [10]. Analysis of the Δ*δ_S_*_−_*_R_* values (Figure 6) according to the Mosher model pointed to an *R* configuration for C-13 of **5**, because H_2_-10, H-11, Me-19, and Me-20 of (*S*)-MTPA ester **5**a were less shielded by the phenyl ring of MTPA products. Therefore, the absolute stereochemistry of **5** was unambiguously established as (1*R*,3*R*,4*R*,13*R*,14*R*,15*S*,7*E*,11*E*)-13-hydroxy-17-methoxy-3,4-epoxycembra-7,11-dien-16,14-olide.

The determination of the configurations of γ-lactone ring fused to 14-membered ring at C-1 and C-14 based on the coupling constant between the lactonic methine protons (^3^*J*_1,14_) is dubious [8]. Notably, all of the *cis*-fused lactones at C-1 and C-14 of cembranolides from the Caribbean gorgonians *Eunicea succinea* and *Eunicea mammosa* for which an X-ray analysis has been performed [23] and all *trans*-fused lactones of durumolides M–Q (**1**–**5**) were confirmed after determination of the ring junction of durumolides A–L and durumhemiketalolides A–C using X-ray data and biogenetic considerations [10–12].

The possibility that **1** and **2** were furnished via Micheal addition [24,25] of sinularolide B [22] in MeOH resulting in addition of a methoxy group to α-methylene-γ-lactone should not be excluded. In order to ascertain **1** and **2**, having an α-methoxymethyl-γ-lactone ring, to be natural or artificial products from sinularolide B through the isolation process, the following experiments were conducted. A solution of sinularolide B was kept at room temperature for 3 days in the presence of Si-60 or RP-18 gel in MeOH. The formation of **1** and **2** were not observed. Apparently, compounds **1** and **2** are natural products. Similarly, compounds **3**–**5**, possessing the same functionality, may also be natural products.

Compounds **1**–**5** in the present study were evaluated *in vitro* for cytotoxicity against P-388, A-459 and HT-29 cancer cell lines using the MTT assay, and antiviral activity against human cytomegalovirus. Preliminary cytotoxic screening revealed that compound **4** exhibited cytotoxicity against P-388 (mouse lymphocytic leukemia) cell line with an ED_50_ of 3.8 μg/mL. Moreover, compound **5** showed significant antiviral activity against human cytomegalovirus with an IC_50_ of 5.2 μg/mL.

## 3. Experimental Section

### 3.1. General Experimental Procedures

Optical rotations were determined with a JASCO P1020 digital polarimeter. Ultraviolet (UV) and infrared (IR) spectra were obtained on a JASCO V-650 and JASCO FT/IR-4100 spectrophotometers, respectively. The NMR spectra were recorded on a Varian MR 400 NMR spectrometer at 400 MHz for ^1^H and 100 MHz for ^13^C or on a Varian Unity INOVA 500 FT-NMR spectrometer at 500 MHz for ^1^H and 125 MHz for ^13^C, respectively. Chemical shifts are expressed in *δ* (ppm) referring to the solvent peaks *δ*_H_ 7.27 and *δ*_C_ 77.0 for CDCl_3_, respectively, and coupling constants are expressed in Hz. ESI-MS were recorded by ESI FT-MS on a Bruker APEX II mass spectrometer. Silica gel 60 (Merck, Germany, 230–400 mesh) and LiChroprep RP-18 (Merck, 40–63 μm) were used for column chromatography. Precoated silica gel plates (Merck, Kieselgel 60 F_254_, 0.25 mm) and precoated RP-18 F_254s_ plates (Merck) were used for thin-layer chromatography (TLC) analysis. High-performance liquid chromatography (HPLC) was carried out using a Hitachi L-7100 pump equipped with a Hitachi L-7400 UV detector at 220 nm together with a semi-preparative reversed-phase column (Merck, Hibar LiChrospher RP-18e, 5 μm, 250 × 25 mm).

### 3.2. Animal Material

The soft coral *L. durum* was collected by hand using SCUBA along the coast reefs offshore from the Dongsha Atoll in June 2007, at a depth of 8 m, and was stored in a freezer for two months until extraction. Identification was kindly verified by Prof. Chang-Feng Dai, Institute of Oceanography, National Taiwan University, Taiwan. A voucher specimen (TS-13) was deposited in the Department of Marine Biotechnology and Resources, National Sun Yat-sen University, Taiwan.

### 3.3. Extraction and Isolation

The frozen soft coral (1 kg) was chopped into small pieces and extracted exhaustively by maceration with fresh acetone for 24 h at room temperature. The quantity of solvent used for each extraction (2 L) was at least three times the amount of the soft coral material used. The acetone extracts were filtered and concentrated under vacuum to yield a brownish oily residue, which was subsequently partitioned between EtOAc and H_2_O. The resulting EtOAc-soluble residue (30 g) was subjected to column chromatography on silica gel using *n*-hexane with increasing amounts of EtOAc, and finally 100% MeOH as elution, to obtain roughly 30 fractions on the basis of ^1^H NMR data and TLC analyses. Fraction 18 (2.16 g) eluted with *n*-hexane–EtOAc (1:8) was subjected to a silica gel column using *n*-hexane–EtOAc mixtures of increasing polarity for elution, to furnish 14 subfractions. A subfraction 18-8 (507 mg) eluted with *n*-hexane–EtOAc (1:2) was fractionated to column chromatography on an ODS column using aq MeOH to afford six subfractions. Subsequently, a subfraction 18-8-1 (47 mg) eluted with MeOH–H_2_O (45:55) was further purified by HPLC (RP-18) using 70% aq MeOH to give **1** (2 mg) and **2** (1 mg). Similarly, **3** (1 mg), and **5** (3 mg) were obtained by separation of a subfraction 18-8-2 (267 mg) eluted with MeOH–H_2_O (55:45) on HPLC (RP-18) using 70% MeOH in H_2_O. Similarly, a subfraction 18-8-4 (46 mg) eluted with MeOH–H_2_O (65:35) was further applied to HPLC (RP-18) using 70% MeOH in H_2_O to provide **4** (2 mg).

Durumolide M (**1**): colorless oil; [α]^25^ _D_ −45 (*c* 0.1, CHCl_3_); IR (KBr) ν_max_ 3394, 2924, 1766, 1449, 1027, 765 cm^−^^1^; ^1^H-NMR (CDCl_3_, 400 MHz) and ^13^C-NMR (CDCl_3_, 100 MHz) data, see Tables 1 and 2; ESI-MS *m/z* 403 [M + Na]^+^ HR-ESI-MS *m/z* 403.2099 (calcd for C_21_H_32_O_6_Na, 403.2096).

Durumolide N (**2**): colorless oil; [α]^25^ _D_ −98 (*c* 0.1, CHCl_3_); IR (KBr) ν_max_ 3436, 2926, 1766, 1386, 1178, 1033 cm^−^^1^; ^1^H-NMR (CDCl_3_, 500 MHz) and ^13^C-NMR (CDCl_3_, 125 MHz) data, see Tables 1 and 2; ESI-MS *m/z* 403 [M + Na]^+^; HR-ESI-MS *m/z* 403.2094 (calcd for C_21_H_32_O_6_Na, 403.2096).

Durumolide O (**3**): colorless oil; [α]^25^ _D_ −94 (*c* 0.1, CHCl_3_); IR (KBr) ν_max_ 3425, 2924, 1773, 1460, 1181, 1035 cm^−^^1^; ^1^H-NMR (CDCl_3_, 500 MHz) and ^13^C-NMR (CDCl_3_, 125 MHz) data, see Tables 1 and 2; ESI-MS *m/z* 445 [M + Na]^+^; HR-ESI-MS *m/z* 445.2199 (calcd for C_23_H_34_O_7_Na, 445.2202).

Durumolide P (**4**): colorless oil; [α]^25^ _D_ −121 (*c* 0.1, CHCl_3_); IR (KBr) ν_max_ 3460, 2926, 1769, 1445, 1181, 1031 cm^−^^1^; ^1^H-NMR (CDCl_3_, 400 MHz) and ^13^C-NMR (CDCl_3_, 100 MHz) data, see Tables 1 and 2; ESI-MS *m/z* 387 [M + Na]^+^; HR-ESI-MS *m/z* 387.2148 (calcd for C_21_H_32_O_5_Na, 387.2147).

Durumolide Q (**5**): colorless oil; [α]^25^ _D_ −99 (*c* 0.2, CHCl_3_); IR (KBr) ν_max_ 3446, 2924, 1773, 1383, 1177, 1031 cm^−^^1^; ^1^H-NMR (CDCl_3_, 400 MHz) and ^13^C-NMR (CDCl_3_, 100 MHz) data, see Tables 2 and 3; ESI-MS *m/z* 387 [M + Na]^+^; HR-ESI-MS *m/z* 387.2146 (calcd for C_21_H_32_O_5_Na, 387.2147).

Preparation of Mosher’s Esters of **5**: In separate NMR tubes, duplicate (1.0 mg) samples of **5** were dissolved in 0.6 mL of pyridine-*d*_5_ and allowed to react for 3 hr at room temperature with (*R*)- and (*S*)-MTPA chloride (one drop) to yield (*S*)-MTPA ester **5a** and (*R*)-MTPA ester **5b**, respectively. Selected ^1^H-NMR (pyridine-*d*_5_, 300 MHz) of **5a**: *δ*_H_ 7.41–7.61 (5H, m, Ph), 5.93 (1H, br d, *J* = 8.9 Hz, H-13), 5.74 (1H, m, H-11), 5.00 (1H, br d, *J* = 9.1 Hz, H-7), 4.44 (1H, t, *J* = 8.9 Hz, H-14), 4.01 (2H, m, H_2_-17), 3.26 (3H, s, OMe), 2.88 (1H, m, H-15), 2.82 (1H, br s, H-1), 2.47 (2H, m, H_2_-10), 2.20 (2H, m, H_2_-6), 1.95 (2H, m, H_2_-2), 1.78 (3H, s, Me-20), 1.54 (3H, s, Me-19), 1.21 (3H, s, Me-18); Selected ^1^H-NMR (pyridine-*d*_5_, 300 MHz) of **5b**: 7.42–7.63 (5H, m, Ph), 5.83 (1H, br d, *J* = 8.4 Hz, H-13), 5.60 (1H, m, H-11), 4.94 (1H, br d, *J* = 8.9 Hz, H-7), 4.50 (1H, t, *J* = 8.9 Hz, H-14), 4.02 (2H, m, H_2_-17), 3.27 (3H, s, OMe), 2.93 (1H, m, H-15), 2.83 (1H, m, H-1), 2.38 (2H, m, H_2_-10), 2.19 (2H, m, H_2_-6), 1.97 (2H, m, H_2_-2), 1.54 (3H, s, Me-20), 1.49 (3H, s, Me-19), 1.16 (3H, s, Me-18).

### 3.4. Cytotoxicity Assay

Cytotoxicity was determined against P-388 (mouse lymphocytic leukemia), HT-29 (human colon adenocarcinoma), and A-549 (human lung epithelial carcinoma) tumor cells using a modification of the MTT colorimetric method. The provision of the P-388 cell line was supported by J. M. Pezzuto, formerly of the Department of Medicinal Chemistry and Pharmacognosy, University of Illinois at Chicago. HT-29 and A-549 cell lines were purchased from the American Type Culture Collection. The experimental details of this assay were carried out according to a previously described procedure [11,26,27].

### 3.5. Anti-HCMV Assay

To determine the effects of natural products upon the HCMV cytopathic effect (CPE), confluent human embryonic lung (HEL) cells grown in 24-well plates will be incubated for 1 h in the presence or absence of various concentrations of tested natural product. Then, cells will be infected with HCMV at an input of 1000 plaque forming units (pfu) per well of a 24-well dish. Antiviral activity is expressed as IC_50_ (50% inhibitory concentration), or the compound concentration required to reduce the virus induced CPE by 50% after 7 days as compared with the untreated control. To monitor the cell growth upon treating with natural products, an MTT-colorimetric assay was employed [28].

## 4. Conclusions

Comparison of cytotoxicity of durumolides M–Q (**1**–**5**) with those of cembranolides previously isolated by our group from *Lobophtum michaelae* [29], showed that methoxylation at C-17 reduced cytotoxicity against the same cancer cell lines.

## Figures and Tables

**Table 1.** ^1^H NMR data for compounds **1**–**3**.

	**1***^a^*	**2***^b^*	**3***^b^*
1	2.28 m	2.41 m	2.40 m
2	1.75 dt (14.8, 2.4) *^c^*	1.97 ddd (14..5, 2.0, 2.0)	2.00 br d (14.5)
	1.67 ddd (14.8, 7.6, 2.4)	1.37 ddd (14.5, 8.0, 2.5)	1.21 ddd (14.5, 8.5, 1.5)
3	2.67 dd (7.6, 2.4)	2.73 dd (8.0, 2.5)	2.69 dd (8.5, 1.5)
5	2.43 m	2.39 m	2.38 m
	1.11 ddd (14.8, 13.2, 3.6)	1.11 ddd (14.5, 13.0, 3.5)	1.08 ddd (15.0, 13.5, 3.5)
6	2.31 m	2.32 m	2.29 m
	2.10 m	2.14 m	2.15 m
7	5.04 dd (7.2, 4.4)	5.08 dd (7.5, 4.5)	5.07 dd (7.0, 4.0)
9	2.39 m	2.35 m	2.37 m
	2.13 m	2.16 m	2.20 m
10	2.55 m	2.51 m	2.53 m
	2.16 m	2.19 m	2.17 m
11	5.45 dd (6.8, 4.0)	5.49 dd (6.8, 3.5)	5.50 dd (6.5, 3.0)
13	3.97 d (8.8)	4.11 d (8.5)	4.08 d (8.5)
14	4.20 dd (9.2, 8.8)	4.06 t (8.5)	4.04 dd (9.0, 8.5)
15	2.86 dt (9.2, 2.8)	2.50 dt (10.5, 2.5)	2.49 dt (9.5, 2.5)
17	3.81 dd (9.2, 2.8)	3.81 dd (10.5, 2.5)	3.81 dd (9.5, 2.5)
	3.73 dd (9.2, 2.8)	3.79 dd (10.5, 2.5)	3.79 dd (9.5, 2.5)
18	3.87 dd (12.0, 6.0)	3.84 d (12.0)	4.44 d (12.5)
	3.57 dd (12.0, 4.8)	3.54 d (12.0)	3.77 m
19	1.62 s	1.62 s	1.64 s
20	1.71 s	1.70 s	1.70 s
17-OMe	3.30 s	3.39 s	3.38 s
18-OAc			2.13 s

a Spectra were measured in CDCl_3_ (400 MHz);

b Spectra were measured in CDCl_3_ (500 MHz);

c *J* values (in Hz) are in parentheses.

**Table 2.** ^13^C NMR data for compounds **1**–**5**.

	**1***^a^*	**2***^b^*	**3***^b^*	**4***^a^*	**5***^a^*
1	40.8	38.6	38.7	40.8	38.7
2	25.0	31.4	31.4	31.0	32.2
3	64.3	63.9	63.1	63.7	63.8
4	62.1	62.2	59.9	62.5	59.5
5	33.3	33.5	32.9	33.3	38.3
6	24.1	24.2	24.3	24.7	24.9
7	124.5	124.5	124.3	124.4	124.6
8	135.0	135.1	135.2	135.3	134.6
9	39.0	38.9	38.9	39.3	38.9
10	24.7	24.7	24.8	23.8	24.8
11	132.0	131.9	132.2	130.2	132.3
12	132.4	132.0	132.0	129.9	131.9
13	82.0	80.9	81.2	43.7	81.4
14	84.1	82.2	81.9	80.1	82.2
15	44.5	49.4	49.3	49.8	49.5
16	176.9	175.4	175.3	176.2	175.6
17	70.0	68.1	67.8	68.5	67.9
18	61.8	61.5	63.7	61.5	16.3
19	15.3	15.2	15.3	15.0	15.2
20	12.2	12.3	12.1	17.1	12.0
17-OMe	59.1	59.3	59.3	59.2	59.3
18-OAc			20.8		
			170.9		

a Spectra were measured in CDCl_3_ (100 MHz);

b Spectra were measured in CDCl_3_ (125 MHz).

**Table 3.** ^1^H NMR data *^a^* for compounds **4** and **5**.

	**4**	**5**
1	2.51 m	2.41 m
2	1.95 ddd (14.4, 4.0, 2.0) *^b^*	1.98 d (14.4)
	1.45 ddd (14.4, 7.2, 2.4)	1.09 m
3	2.90 dd (7.2, 4.0)	2.52 m
5	2.37 m	2.08 m
	1.21 ddd (14.8, 12.8, 4.0)	1.14 ddd (11.6, 10.4, 2.4)
6	2.33 m	2.29 m
	2.15 m	2.11 m
7	5.09 dd (6.4, 4.4)	5.05 br d (9.6)
9	2.29 m	2.35 m
	2.13 m	2.17 m
10	2.26 m	2.54 m
	2.13 m	2.17 m
11	5.17 t (7.6)	5.50 dd (7.6, 2.8)
13	2.54 m	4.08 d (8.8)
	2.45 m	
14	4.13 ddd (10.0, 8.4, 3.2)	4.03 dd (8.8, 8.4)
15	2.48 m	2.49 t (2.8)
17	3.81 dd (9.6, 3.2)	3.83 dd (9.6, 2.8)
	3.77 dd (9.6, 3.2)	3.79 dd (9.6, 2.8)
18	3.85 dd (12.0, 5.2)	1.21 s
	3.55 dd (12.0 5.2)	
19	1.60 s	1.63 s
20	1.68 s	1.72 s
17-OMe	3.39 s	3.39 s

a Spectra were measured in CDCl_3_ (400 MHz);

b *J* values (in Hz) are in parentheses.
